# Temporally constrained ICA with threshold and its application to fMRI data

**DOI:** 10.1186/s12880-018-0300-6

**Published:** 2019-01-17

**Authors:** Zhiying Long, Zhi Wang, Jing Zhang, Xiaojie Zhao, Li Yao

**Affiliations:** 1 State Key Laboratory of Cognitive Neuroscience and Learning & IDG/McGovern Institute for Brain Research, Beijing Normal University, Beijing, 100875 China; 2 School of Information Science & Technology, Beijing Normal University, Beijing, China

**Keywords:** ICA, fMRI, Temporally constrained ICA, FastICA, Threshold, Task-related component

## Abstract

**Background:**

Although independent component analysis (ICA) has been widely applied to functional magnetic resonance imaging (fMRI) data to reveal spatially independent brain networks, the order indetermination of ICA leads to the problem of target component selection. The temporally constrained independent component analysis (TCICA) is capable of automatically extracting the desired spatially independent components by adding the temporal prior information of the task to the mixing matrix for fMRI data analysis. However, the TCICA method can only extract a single component that tends to be a mix of multiple task-related components when there exist several independent components related to one task.

**Methods:**

In this study, we proposed a TCICA with threshold (TCICA-Thres) method that performed TCICA outside the threshold and performed FastICA inside the threshold to automatically extract all the target components related to one task. The proposed approach was tested using simulated fMRI data and was applied to a real fMRI experiment using 13 subjects. Additionally, the performance of TCICA-Thres was compared with that of FastICA and TCICA.

**Results:**

The results from the simulation and the fMRI data demonstrated that TCICA-Thres better extracted the task-related components than TCICA. Moreover, TCICA-Thres outperformed FastICA in robustness to noise, spatial detection power and computational time.

**Conclusions:**

The proposed TCICA-Thres solves the limitations of TCICA and extends the application of TCICA in fMRI data analysis.

**Electronic supplementary material:**

The online version of this article (10.1186/s12880-018-0300-6) contains supplementary material, which is available to authorized users.

## Background

Functional magnetic resonance imaging (fMRI) is a powerful technique to indirectly reveal the neural representation of various cognitive processes. Both univariate methods and multivariate methods have been widely applied to fMRI data analysis. Because the multivariate methods do not treat each voxel independently and consider the relationships between voxels, they have attracted more and more attention in fMRI data analysis compared to the univariate methods. Among the various multivariate methods, independent component analysis (ICA), a kind of blind source separation method, is powerful at detecting independent brain networks from fMRI data [1]. Although both spatial ICA and temporal ICA can be used to extract the spatially independent components and temporally independent components, respectively, spatial ICA is much more widely used than temporal ICA [2].

ICA is a data-driven method that can separate the intrinsic independent sources from data without any prior information. The semiblind ICA methods and constrained ICA (CICA) have been proposed to improve the performance of ICA by adding some prior information. The semiblind ICA imposes regularization on certain estimated time courses using the paradigm information [3, 4]. In contrast to semiblind ICA, CICA can automatically estimate the target components in a predefined order by adding constraints to the classical ICA algorithm [5, 6]. The prior information can be added to either the source matrix [5] or the mixing matrix [7] as constraints. CICA has been applied to the fMRI data analysis in several studies. In Lu’s study, the temporally independent component that was related to the task was estimated from fMRI data by CICA using temporal constraints on the source matrix without separating all of the sources [5]. Moreover, the desired spatially independent components were extracted from fMRI data by CICA using spatial constraints on the sources matrix [8]. To improve the convergence of CICA, Wang et al. (2011) proposed learning-rate-free CICA algorithms that were applied to separate spatially independent component from fMRI data using a temporal constraint on the mixing matrix [9]. Recently, Wang (2014) proposed a temporally and spatially constrained ICA (TSCICA) by using the temporal constraints on the mixing matrix and spatial constraints on the source matrix to extract the desired spatially independent components from fMRI data [10]. Rodriguez et al. (2015) proposed general nonunitary constrained ICA, which they applied to extract the spatially independent component from complex-valued fMRI data by using the temporal constraint on the mixing matrix [11].

Task fMRI data generally contain one or more spatially independent components that are related to the same task [2]. It should be noted that CICA with the temporal constraint as used in the previous studies could only extract one task-related component and failed to extract all the spatial components that were related to the same task from the task fMRI data in the above studies. However, one or more task-related spatial components can be separated by spatial ICA [2]. For task fMRI data, the temporal prior information is usually derived from the convolution of the task paradigm with the hemodynamic response (HRF). If more than one spatial component is related to one task, the ICA contrast function will contain more than one extreme point that are close to the temporal reference of the task. When temporal CICA (TCICA) adds the temporal constraint to the cost function of ICA using the Lagrange multiplier method during fMRI data analysis, the optimal surface of the cost function is changed to retain an extreme point close to the temporal reference and remove all the irrelevant extreme points so that the desired task-related component can be extracted. Because the time course of each task-related component is highly correlated with the temporal reference of the task, the target component extracted by TCICA may mix several task-related components. Accordingly, TCICA is not able to fully extract all the spatially independent components that are related to one task from fMRI data. Although our previous study [10] proposed a temporally and spatially constrained ICA method, TSCICA fails to work when the spatial prior information is unavailable. Moreover, TSCICA cannot separate all the components that are related to the same task because it is difficult to obtain the spatial prior information of all the task-related components. Therefore, the applications of TCICA and TSCICA are largely limited.

This study aimed to apply ICA to task-related activity by adding some constraints in the mixing matrix, which should lead to increased chance of detectability and a gain in computation time. We proposed the TCICA with threshold (TCICA-Thres) method, which was able to automatically extract all the spatially independent components related to one task without estimating all the independent components from fMRI data. The basic idea of TCICA-Thres is to perform TCICA outside the threshold to remove all the irrelevant extreme points and perform FastICA inside the threshold to keep all the extreme points close to the temporal reference. Using simulated and real fMRI experiments that contained one task, we investigated the robustness, the feasibility and the stability of the proposed method and compared TCICA-Thres with FastICA and TCICA. The results from both the simulated and real fMRI experiments demonstrated that TCICA-Thres extracted all the components that were related to a task, while TCICA could not. Moreover, TCICA-Thres showed better performance than FastICA in both detection power and computation time. It should be noted that this study used spatial ICA for the TCICA-Thres, FastICA and TCICA methods.

## Methods

### TCICA

The spatial ICA model of fMRI data can be expressed by (1).

1 $$ \mathbf{X}=\mathbf{AS} $$

where **X**_K × V_ is the observed fMRI signal data, **A**_K × C_ is the mixing matrix and **S**_C × V_ is the source matrix. K represents the number of scans, V represents the number of voxels, and C represents the number of total independent components. ICA seeks an unmixing matrix **W**.

The temporal CICA (TCICA) method is modeled as the following constrained optimization problem [7]:

2 $$ \mathit{\operatorname{Max}}J(y)\kern0.5em ={\left\{E\left[f(y)\right]-E\left[f(v)\right]\right\}}^2\kern0.5em s.\kern0.5em t.\kern0.5em g(w)=\varepsilon \left(w,{r}_t^{\hbox{'}}\right)-\xi \kern0.5em \le \kern0.5em 0,\kern0.5em h(y)=E\left\{{y}^2\right\}-1=0 $$

where *J*(*y*) denotes the contrast function of the FastICA algorithm; *ε*(*w*, *r*_*t*_^′^) is the closeness measure between the unmixing vector *w* and *r*_*t*_^′^, where *r*_*t*_^′^ is the transformation of the temporal reference *r*_t_ into the unmixing space; and ξ is a threshold that can distinguish one desired unmixing vector *w* from the others. The temporal reference signal *r*_t_ can be constructed from the convolution of the task paradigm with HRF.

### TCICA-Thres

Assume the total number of source signals is M and that there are L task-related components (1 ≤ L < M) whose time courses are highly correlated with the temporal reference *r*_t._ Because the extreme points close to the temporal reference usually correspond to the task-related components, the basic idea of TCICA-Thres is to keep all the extreme points of the task-related components by using the FastICA contrast function inside the threshold and the TCICA contrast function outside the threshold. The proposed TCICA-Thres method automatically extracts the L desired components from the observed data in a predefined order instead of estimating all of the M components, as standard ICA does.

For the negentropy J(*y*) in eq. (2), *v* is a Gaussian variable and is unrelated to the variable y. The first-order derivative of J(*y*) is 2α(E{*f*(*y*)} − E{*f*(*v*)}). Therefore, the maxima of Negentropy *J*(*y*) = *α*[*E*{*f*(*y*)} − *E*{*f*(*v*)}]^2^ are obtained at certain optima of *E*{*f*(*y*)}, and the objective function of FastICA can be simplified as *J*(*y*) = *E*{*f*(*y*)} [12]. Therefore, the proposed TCICA-Thres method can be formulated in the framework of CICA and FastICA:

3 $$ \operatorname{maximize}\kern0.5em J(y)=E\left\{f(y)\right\} $$

4 $$ \mathrm{subject}\ \mathrm{to}\kern.5em g\left(w,{r}_t^{\hbox{'}}\right)=\varepsilon \left(w,{r}_t^{\hbox{'}}\right)-\xi \left\{{}_{=0,\kern0.5em \rho \kern0.5em \left(w,{r}_t^{\hbox{'}}\right)\kern0.5em > threshold}^{\le 0,\kern0.62em \rho \kern0.5em \left(w,{r}_t^{\hbox{'}}\right)\kern0.5em \le threshold}\right. $$

5 $$ h(w)=E\left\{{w}^2\right\}-1=0 $$

where $\rho \left(w,{r}_t^{\hbox{'}}\right)$ is the correlation coefficient between the unmixing vector *w* and *r’*_*t*_ , where *r’*_*t*_ is the transformation of *r*_*t*_ into the unmixing space. If $\rho \left(w,{r}_t^{\hbox{'}}\right)$ computed in (4) is below the *threshold*, the inequality constraint *g* works, and the estimated *w* will be corrected to be close to the predictive model. Otherwise, the correction term *g* does not work. Based on the constraints of the eq. (4) and (5), TCICA-Thres can estimate the optimal solution of eq. (3) using Lagrange multipliers. The corresponding augmented Lagrange function *L* of TCICA-Thres is given by:

6 $$ L=J(y)-G\left(w,{r}_t^{\hbox{'}},\mu \right)-H\left(w,\lambda \right) $$

where

7 $$ G\left(w,{r}_t^{\hbox{'}},\mu \right)=\Big\{{}_{0,\kern11.5em \rho \left(w,{r}_t^{\hbox{'}}\right)> threshold}^{S_c\times \frac{1}{2_{\gamma }}\kern0.5em \left[{\max}^2\left\{\mu +\gamma g\left(w,{r}_t^{\hbox{'}}\right),0\right\}-{\mu}^2\right]\kern1em \rho \left(w,{r}_t^{\hbox{'}}\right)\kern0.5em \le threshold}\operatorname{} $$

8 $$ H=\lambda \left[E\left({w}^2\right)-1\right] $$

$G\left(w,{r}_t^{\hbox{'}},\mu \right)$ transforms the original inequality constraint of the temporal reference signal into equality constraint; μ and λ are the positive Lagrange multipliers that are the weights of the temporal inequality and equality constraint, respectively; γ is the positive penalty parameter;${\mathrm{s}}_{\mathrm{c}}=1/\left(1+\frac{1}{threshold-\rho}\right)$ is a smoothing function that ensures the smooth connection of the constraint function *G* at the threshold point.

The gradient descent learning algorithm, which was used to solve the optimization problem, and the procedure of the TCICA-Thres algorithm are presented in the Appendix of the Additional file 1.

### Simulation of single-subject analysis

In this section, the simulated fMRI experiments were performed to investigate the robustness and the feasibility of the proposed TCICA-Thres method and further compare the performance of TCICA-Thres with FastICA at different noise levels. Moreover, the TCICA-Thres methods using different temporal references were applied to investigate the robustness of TCICA-Thres to the temporal references.

Principal component analysis (PCA) was applied to each simulated dataset to reduce dimension with 99.9% of the total variance of the mixed signals retained prior to ICA. This ensured all the informative components were included. The nonlinear function *f*(·) in Eq. (4) and Eq. (12) used *f*(·) = y^3^/3. For the TCICA-Thres method, the learning rate η was set to 10^− 4^ × (0.5 × cos(π × (k-1)/99) + 0.5)^n^, where n is set to 2 and k is the iterative step. The use of this value for n of the learning rate was validated in the following simulation. Generally, the learning rate η is set as a fixed value. The learning rate in this study decreased gradually with the increase in the iterative step to ensure stable convergence. For the TCICA-Thres method, the *threshold* of ρ was set to 0.5, the penalty parameter γ was set to 0.1 × 4^(k-1)^ [9], and the Lagrangian multipliers μ and λ were initialized to 1 [10]. The correlation was used as the closeness measure such that $\varepsilon \left({w}_i,{r}_{ti}^{\hbox{'}}\right)=-E\left\{{w}_i,{r}_{ti}^{\hbox{'}}\right\}$. According to our previous study [10], the threshold ξ was initialized to 0.9 and was adjusted according to the correlation coefficient of the estimated *w*_*i*_ and ${r}_{ti}^{\hbox{'}}$ during each iteration. The termination criterion was set to ||Δw|| < 10^− 4^ for TCICA-Thres and FastICA. A maximum of 100 iterations was allowed for each ICA decomposition run of TCICA-Thres and FastICA. The core FastICA algorithm was downloaded from the internet [13]. The TCICA-Thres was developed in MATLAB (MathWorks, Natick, MA, USA) based on the FastICA algorithm. Receiver operating characteristic (ROC) analysis was applied to compare the spatial detection power of the different methods.

#### Generation of simulated data

**Fig. 1:**
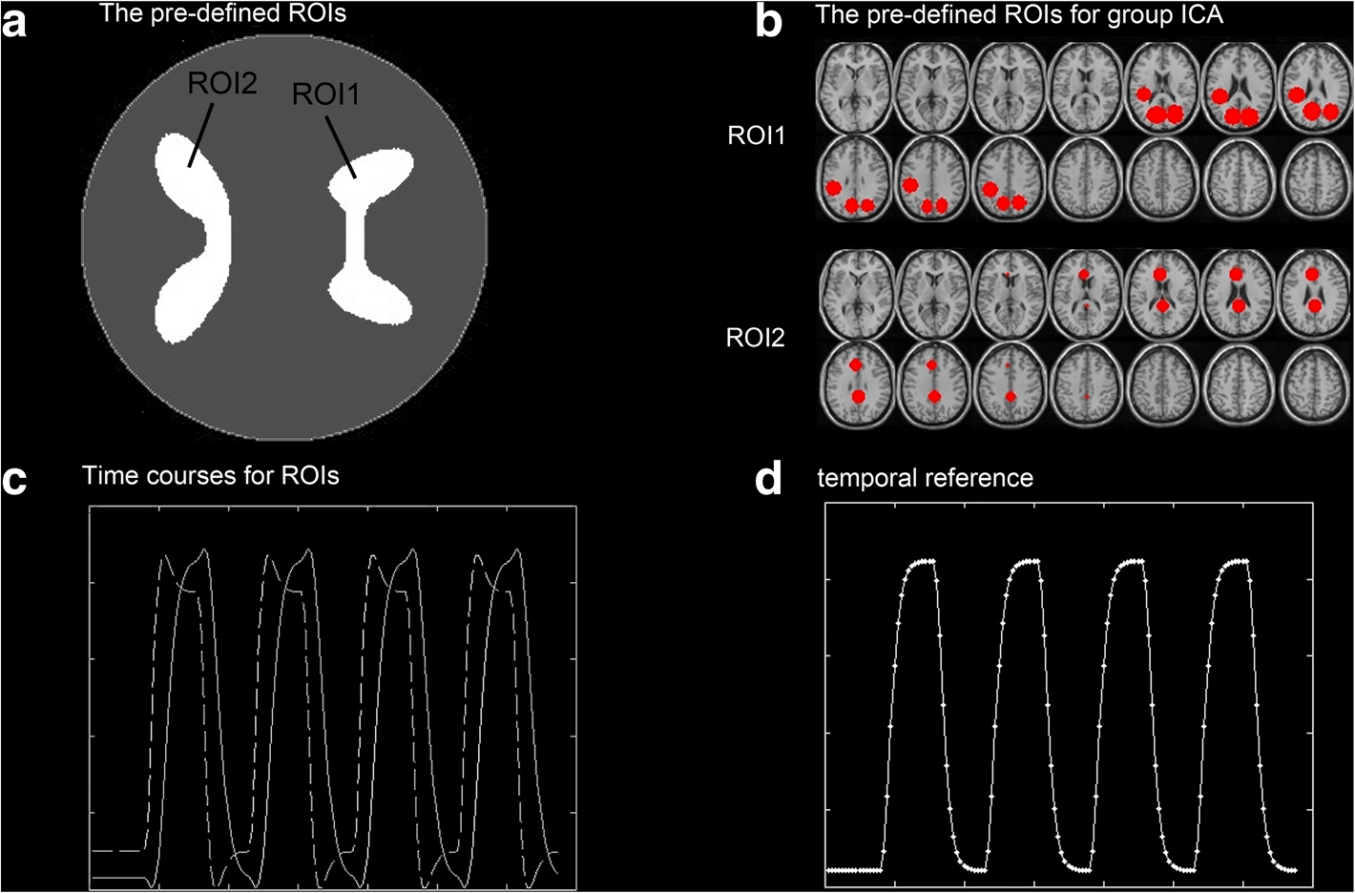
Generation of simulated data. (**a**) The predefined ROI for simulation of single-subject analysis. (**b**) The predefined ROI for simulation of multi-subject analysis. (**c**) The time courses of simulated fMRI responses that are added to the two ROIs. (**d**) The time course of temporal reference used in the simulation of multi-subject analysis

The SimTB toolbox [14] was used to generate fMRI-like simulated datasets with different contrast-to-noise ratios (CNRs). Each dataset consisted of 200 × 200 pixels. We assumed that the simulated experiment included one task that induced two spatial components. The entire 270-s session consisted of four 30-s task blocks and five 30-s rest blocks. In reality, many complicated factors may cause the time courses of the components that are related to one task to be different. For simplicity, we further assumed that the time courses driving different spatial components that were related to the same task only differed in the shape of HRF in the simulation. The spm_hrf function in the software SPM8 (Statistical Parametric Mapping) [15] was used to generate the HRFs. Two ROIs were generated by using the SimTB toolbox (see Fig. 1A). The activated regions of the first and second task-related component were supposed to be the ROI1 and ROI2, respectively. The simulated fMRI responses of the two ROIs were generated by convolving the task paradigm with the two different HRFs (see Fig. 1C). The seven vector parameters of the two HRFs of ROI1 and ROI2 were set to P2 = [14 8 2 2 6 0 32] and P1 = [6 16 1 1 6 0 32], respectively. Each dataset contained Rician noise with a specific CNR. The CNR varied from 0.05 to 0.15, with an increment of 0.01. Ten simulated datasets were generated for each noise level, and a total of 110 simulated datasets were produced.

#### Generation of temporal references

To investigate the robustness of the proposed method to the accuracy of the temporal reference, thirteen temporal references that were derived from the convolution of the experimental paradigm with different HRFs were generated. The correlation coefficients between each of the thirteen temporal references and the true time course underlying the second component were 0.3199, 0.3335, 0.3527, 0.3779, 0.4095, 0.4469, 0.4891, 0.5542, 0.6165, 0.7069, 0.7867, 0.8548 and 0.9248, respectively. Here, we called the temporal reference set TRef.

#### Robustness to the noise magnitude

In this simulation, one temporal reference with correlation coefficient (CC) =0.8548 from TRef was considered. TCICA-Thres was applied to each dataset to automatically extract the two desired task-related components. Additionally, FastICA was applied to each dataset, and the task-related components were selected by using the temporal correlation between the time course of each component and the temporal reference. The two components with the highest temporal correlation coefficients were selected as the task-related components. Meanwhile, the ROC area of each task-related component was obtained for each TCICA-Thres/FastICA application. To investigate how the noise level affected the performance of TCICA-Thres and FastICA, the mean ROC areas across 10 datasets were calculated at each noise level. Moreover, the difference of the ROC area between TCICA-Thres and FastICA at each noise level was examined by the nonparametric Wilcoxon test for paired samples.

#### Determination of the parameter of the learning rate

All 110 simulated datasets with different CNRs were used in this simulation. To avoid an endless loop, the learning rate in the TCICA-Thres algorithm was reduced as the iterative step increased. The learning rate in TCICA-Thres was set as 10^− 4^ × (0.5 × cos(pi×(k-1)/99) + 0.5)^n^, where k is the iteration step. The parameter *n* varied from 1 to 12, with an increment of 1. For a particular *n*, TCICA-Thres that used the temporal reference with CC = 0.85 from TRef as a constraint was applied to each dataset to automatically estimate the two task-related components. The ROC area of each TCICA-Thres processing was calculated. For each n, the mean of 110 ROC areas of each task-related component was calculated, and the sum of the mean ROC areas of the two task-related components was obtained.

#### Robustness to temporal reference

In this experiment, the 20 simulated datasets with high noise level (CNR = 0.08) and low noise level (CNR = 0.14) were used to investigate the impacts of different temporal references on the performance of TCICA-Thres. The TCICA-Thres method that used the thirteen temporal references from TRef in sequence was applied to each dataset to automatically extract the desired two task-related components. For each temporal reference, the mean value of 10 ROC areas across the 10 simulated datasets at each of the two CNR levels was calculated to evaluate the stability of the proposed method.

#### Activation pattern comparison for TCICA-Thres, TCICA, FastICA and GLM

One simulated dataset with CNR = 0.1 was used in this experiment to investigate the differences between TCICA-Thres, TCICA, FastICA and GLM. TCICA-Thres and TCICA that used the temporal reference with CC = 0.85 from TRef as the temporal constraint were applied to the simulated dataset to extract the task-related components. FastICA was directly applied to the simulated datasets. After the TCICA-Thres, TCICA and FastICA processing, the task-related components were transformed into Z score. The activated regions were determined by selecting the voxels with Z scores higher than 2. Moreover, GLM analysis in SPM8 was applied to the dataset by using the temporal reference with CC = 0.85 from TRef as the regressor.

A one-sample t-test was performed to estimate the activated brain regions. The significance level of the t-test was set to *p* < 0.001 without correction.

### Simulation of multi-subject analysis

For multi-subject analysis, temporal concatenation was integrated with TCICA-Thres. In this section, a human fMRI resting data-based simulation was performed to examine the feasibility of the group TCICA-Thres method.

#### Participants

Ten right-handed college students (five females, five males; age: 22.5 ± 3.1 years) with normal vision took part in the experiment. Subjects relaxed with their eyes closed and remained still for 270 s during the entire fMRI scan. All participants gave written consent according to the guidelines set by the MRI center of Beijing Normal University. The experiment was approved by the Institutional Review Board of the State Key Laboratory of Cognitive Neuroscience and Learning in BNU.

#### Imaging parameters

Brain scans were performed using a 3.0-T Siemens whole-body MRI scanner. A single-shot T2*-weighted gradient-echo, echo planar imaging (EPI) sequence was used for functional imaging acquisition with the following parameters: repeated time (TR) = 2000 ms, echo time (TE) = 30 ms, flip angle = 90°, field of view (FOV) = 200 × 200 mm, matrix = 64 × 64, and slice thickness = 3.6 mm. Thirty-three axial slices parallel to the line connecting the anterior and posterior commissures were obtained in an interleaved order to cover the entire cerebrum and part of the cerebellum.

#### Pre-processing

Each participant’s functional images were first realigned to remove head motion. Then, the images were spatially normalized into the standard MNI template space and resliced into 3 × 3 × 4 mm voxels. Finally, the normalized images were smoothed by an 8 × 8 × 8 mm^3^ full width at half-maximum (FWHM) Gaussian kernel.

#### Generation of simulated data

The preprocessed fMRI resting datasets of 10 subjects were used to generate the simulated datasets. The same experimental paradigm as the above simulated experiments were used in this simulation. Two spatial components were assumed to be related to the same task. The activated regions of the first and second task-related components were assumed to correspond to ROI1 and ROI2, respectively (see Fig. 1B). The ROIs were constructed using the 3D ROI tool in MRIcro software. The time course activating the ROI1/ROI2 was produced in the same way as the above simulation (see Fig. 1C). A total of 10 simulated data sets, one for each subject with different SNRs, were generated. Considering the size and shape variation of the activated regions across subjects, 90% of voxels within each ROI of each subject were randomly selected as activated. All ten datasets had the same minimum signal change (0.3%) relative to the mean intensity value of the individual voxel. The maximum signal change of each dataset randomly varied from 1.0 to 1.1%.

#### Group ICA processing

The group TCICA-Thres, group FastICA and group TCICA methods were applied to the simulated data of ten subjects. The number of ICA components was set to 26 according to the minimum description length (MDL) criteria. Each subject’s data was reduced to 26 time points using PCA, and the reduced data of all subjects were concatenated together in the temporal space. The aggregate data set was further reduced to the dimension of 26 using PCA. The reduced data were then decomposed by TCICA-Thres and TCICA to automatically extract the task-related components and by FastICA to extract all the ICs. The temporal reference that was used in the TCICA-Thres and TCICA methods are shown in Fig. 1D. After Thres-ICA/TCICA/FastICA processing, the individual time courses and spatial maps for every subject’s functional data were reconstructed by back reconstruction. The mean time course of each independent component that was separated by FastICA was calculated across the 10 subjects. To identify the task-related components for FastICA, the temporal correlation between the mean time course of each component and the temporal reference was calculated. The two components with the highest temporal correlation coefficients were selected as the task-related components. Moreover, the subsequent group analysis of the task-related components that were estimated by TCICA-Thres and FastICA were conducted using the one-sample t-test in the software SPM8 to identify the brain regions that were significantly engaged in each task-related component.

GLM in SPM8 was applied to each subject’s dataset by using the task paradigm as the regressor. After the individual GLM analysis, a random-effects model was applied to perform the group analysis. The brain regions that were significantly activated by each task were estimated by using the one-sample T test. The statistical results for TCICA-Thres, FastICA, TCICA and GLM were corrected for multiple comparisons via a family-wise error (FWE) at *p* < 0.05.

### The real fMRI experiment

In this section, a real fMRI experiment was performed to further demonstrate the feasibility of the proposed method and to compare the performances of TCICA-Thres and FastICA.

#### Participants

Thirteen volunteer participants (seven females and six males, mean age 22 ± 1 years) participated in the fMRI experiment. All of the subjects were right-handed and had normal vision. The handedness of each subject was confirmed in focused interviews using the Edinburgh inventory [16]. All participants gave written consent according to the guidelines set by the MRI center of Beijing Normal University. The experiment was approved by the Institutional Review Board of the State Key Laboratory of Cognitive Neuroscience and Learning in BNU.

#### Imaging parameters

Brain scans were performed at the MRI Center at Beijing Normal University using a 3.0-T Siemens whole-body MRI scanner. A single-shot T2*-weighted gradient-echo EPI sequence was used for functional imaging acquisition using the parameters TR/TE/flip angle = 2000 ms/30 ms/90°; acquisition matrix = 64 × 64; FOV = 240 mm; and slice thickness = 3.6 mm with interslice gap = 0.6 mm. Thirty-three axial slices parallel to the AC-PC line were obtained in an interleaved order to cover the entire cerebrum and cerebellum.

#### Experimental design

The entire 270-s session consisted of five 30-s rest blocks that were alternated with four 30-s task blocks. During the rest blocks, the subjects relaxed with eyes opened. During each task blocks, 15 object pictures were displayed in the center of the screen. The subjects were required to press the button with their left middle finger if any picture repeated itself consecutively and press the button with their right middle finger if any picture did not repeat itself. Each stimulus was presented for 500 ms and followed by a 1500 ms blank screen.

#### Preprocessing

The same preprocessing steps as the simulation of multi-subject analysis were applied to the fMRI data of each subject.

#### Data analysis

The temporal reference was derived from the convolution of the task paradigm with the HRF that was generated by SPM using the default parameters. The number of ICA components was set to 28 according to the MDL. Both group Thres-ICA and TCICA were applied to identify the brain regions that were engaged in each task-related component using the same processing steps as the simulation of multi-subject analysis.

The GLM analysis was also applied to each dataset after processing by a high-frequency filter and by global scaling using the software SPM8. After the individual GLM analysis, a random-effects model was applied to perform the group analysis. The brain regions that were engaged in each task were estimated by using the one-sample T test. All of the statistical results of the ICA methods and GLM were corrected for multiple comparisons via an FWE at *p* < 0.05.

To compare the stability of TCICA-Thres and FastICA, we used a quantitative evaluation of the compactness of the clusters of independent component estimation. For each subject, the TCICA-Thres and FastICA estimation were repeated 20 times, and the cluster quality index of each method was calculated using the ICASSO software package. A cluster quality index close to 1 indicates that the result is consistent and stable.

## Results

### Simulation of single-subject analysis

#### Robustness to noise magnitude

**Fig. 2:**
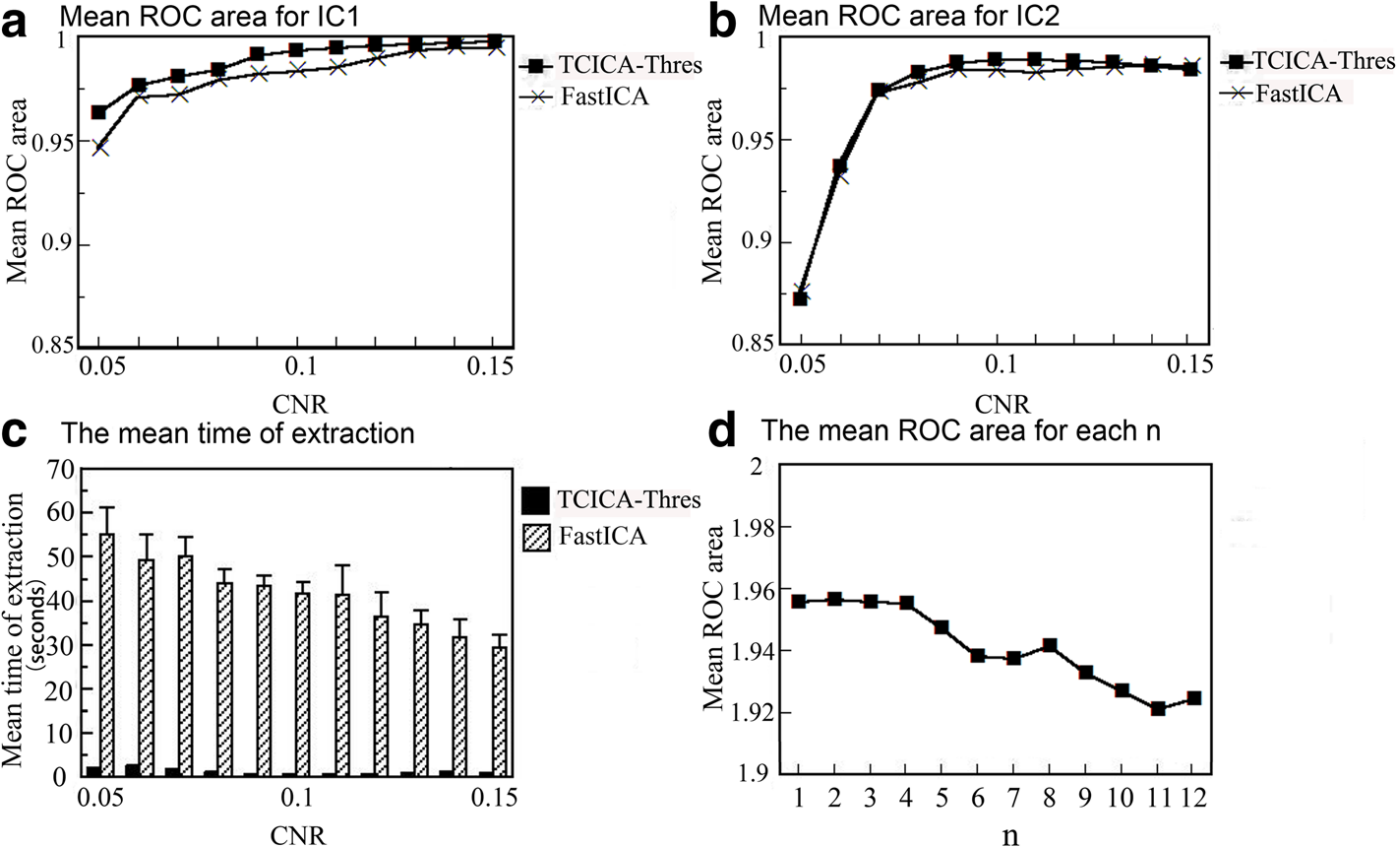
Results of simulation of single-subject analysis. (**a**) The variation of mean ROC area of IC1 extracted by Thres-TCCIA and FastICA with CNR. (**b**) The variation of mean ROC area of IC2 extracted by Thres-TCCIA and FastICA with CNR. (**c**) The mean extraction time of Thres-TCCIA and FastICA at various CNR levels. (**d**) The variation of the sum of the mean ROC areas of IC1 and IC2 with n

The mean ROC areas of the two task-related components at various noise levels are shown in Fig. 2A and B. It can be seen that the mean ROC areas of both TCICA-Thres and FastICA increased with the increase in the CNR. For IC1, TCICA-Thres exhibited higher ROC areas than FastICA at all noise levels (see Fig. 2A). For IC2, TCICA-Thres showed larger ROC areas than FastICA at the medium noise levels and similar ROC areas at the low and high noise levels (see Fig. 2B). Moreover, the mean computation time of ICA processing at the various noise levels are shown in Fig. 2C. It can be seen that TCICA-Thres took much less time to extract the task-related components than FastICA at all the noise levels.

#### Determination of the parameter n of the learning rate

The variation of the sum of the two ICs’ ROC areas with n is presented in Fig. 2D. The results showed that the sum of the mean ROC areas of the two ICs was the greatest when n was equal to 2. Thus, 2 was selected as the optimal value of n in TCICA-Thres for both the entire simulation and the real fMRI experiment.

#### Robustness to temporal reference

**Fig. 3:**
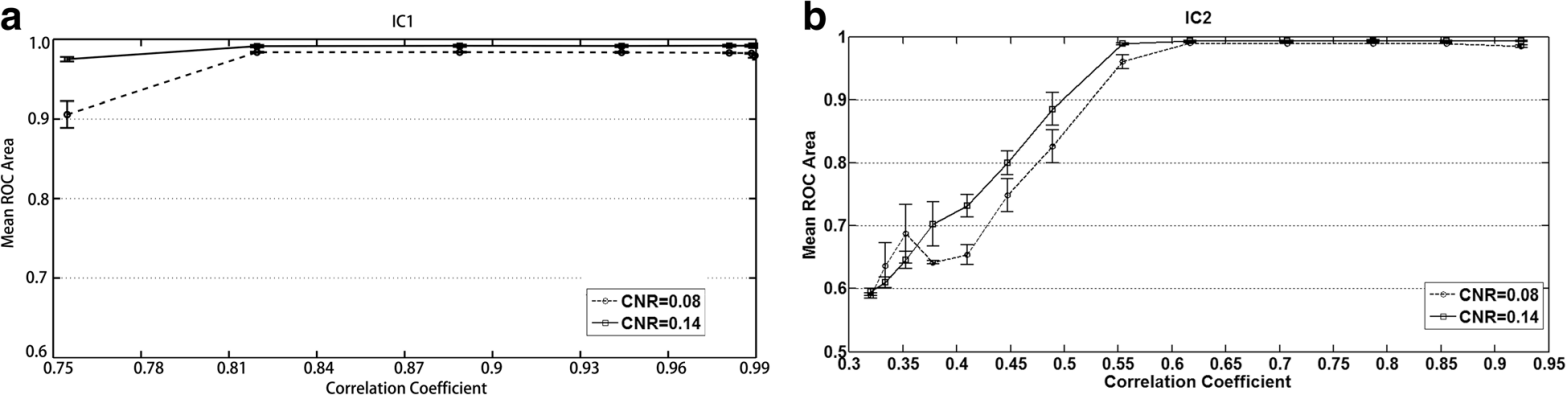
The variation of the mean ROC with the correlation coefficient of temporal reference in the case of CNR = 0.08(**a**) and CNR = 0.14 (**b**) for IC1 and IC2. CC represents correlation coefficient between the temporal reference and the time course of IC2

Figure 3 shows the mean ROC areas of the two ICs that were extracted by TCICA-Thres for various temporal references. It can be seen that the mean ROC area of IC2 increased with the increase in the CC between the temporal reference and the time course underlying IC2 for both the low noise level (CNR = 0.14) and the high noise level (CNR = 0.08) (see Fig. 3B). When CC was below 0.55, the mean ROC area of IC2 dropped fast. However, the mean ROC of IC1 showed slight variation when CC between the temporal reference and the time course underlying IC2 increased. Because the correlation between each temporal reference in TRef and the true time course underlying IC1 was 0.9884, 0.9896, 0.9911, 0.9927, 0.9944, 0.9957, 0.9960, 0.9917, 0.9809, 0.9441, 0.8888, 0.8196 and 0.7548, the mean ROC area of IC1 decreased for the last temporal reference.

#### Comparison of TCICA-Thres, TCICA, FastICA and GLM

**Fig. 4:**
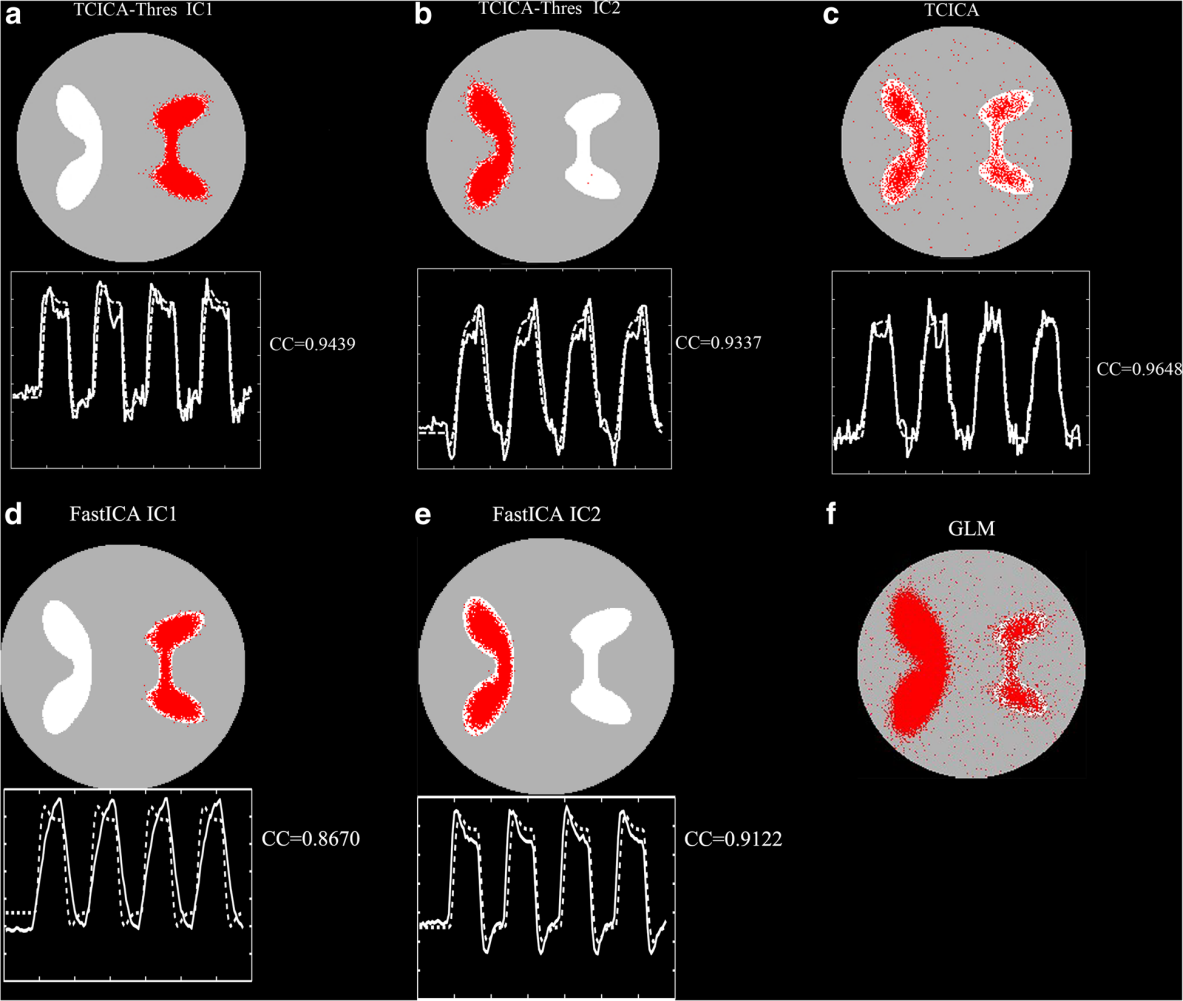
Comparison of Thres-TCCIA, TCICA, FastICA and GLM. (**a**-**b**) The spatial activation and the corresponding time courses (the solid line) of IC1 (**a**) and IC2 (**b**) estimated by TCICA-Thres. (**c**) The spatial activation and the corresponding time courses (the solid line) of the task-related component estimated by TCICA. (**d**-**e**) The spatial activation and the corresponding time courses (the solid line) of IC1 (**d**) and IC2 (**e**) estimated by FastICA. (**f**) The activation detected by GLM

Figure 4 shows the activated regions that were estimated by GLM and the activated regions of the task-related components that were extracted by TCICA-Thres, TCICA and FastICA. For TCICA-Thres and FastICA, the activated region of IC1/IC2 largely overlapped with the ROI1/ROI2, and the time course of IC1/IC2 was highly correlated with the temporal response that was added to the ROI1/ROI2 (see Fig. 4A, B, D and E). Thus, the two task-related components were successfully extracted by Thres-ICA and FastICA from the simulated dataset. Compared to FastICA, Thres-ICA detected larger activated regions for IC1 and IC2. In contrast, the activated regions of the task-related component that was extracted by TCICA contained both ROI1 and ROI2 (see Fig. 4C), which indicated that TCICA merged the two task-related components into one IC and failed to fully separate the two task-related components. Because GLM can detect all the regions that were engaged in the same task, the activated regions included both ROI1 and ROI2 (see Fig. 4F). Moreover, compared to TCICA and GLM, TCICA-Thres and FastICA detected less false activation than TCICA and GLM.

### Simulation of multi-subject analysis

**Fig. 5:**
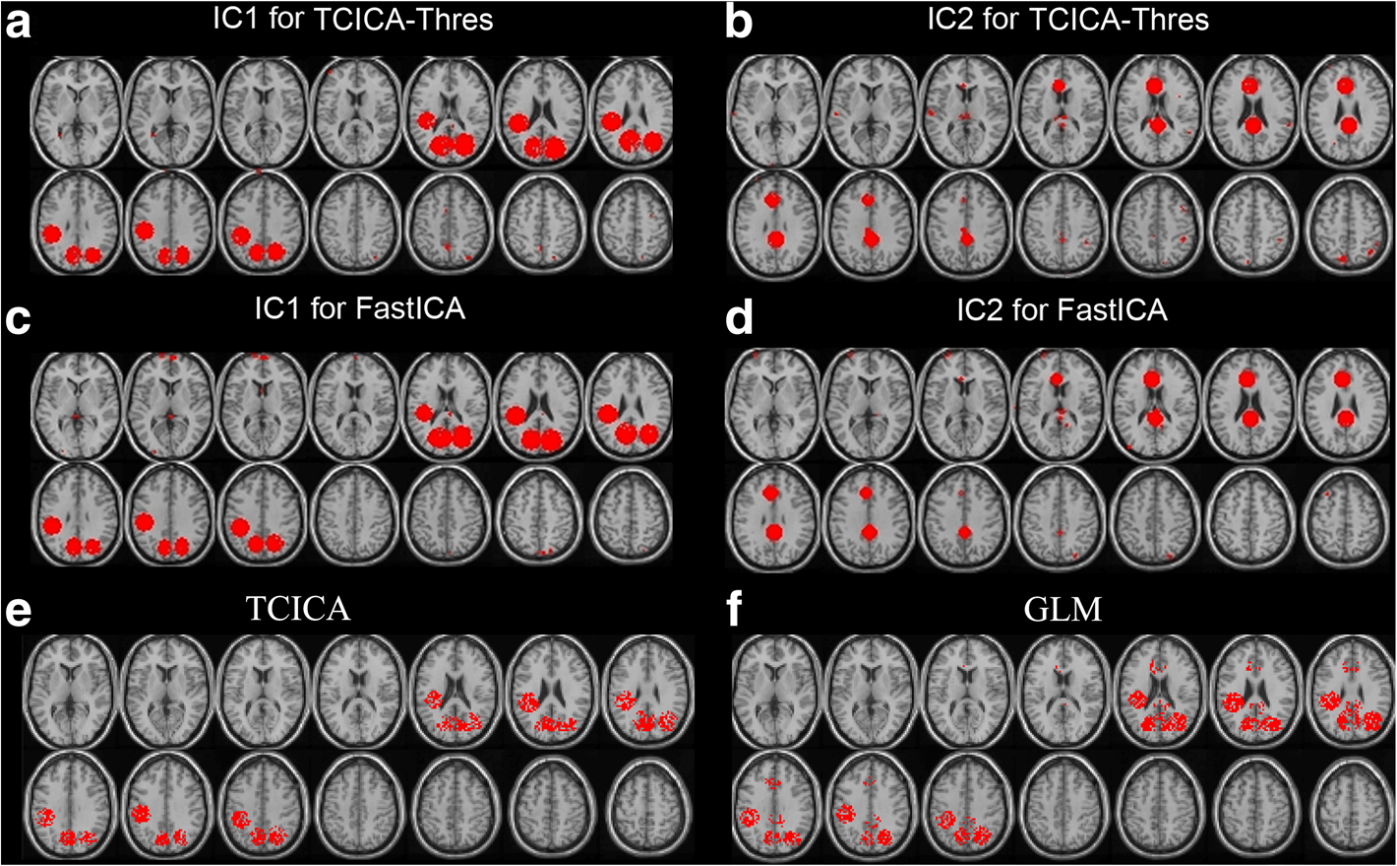
Results of simulation of multi-subject analysis. (**a**-**b**) The spatial activation of IC1 (**a**) and IC2 (**b**) estimated by TCICA-Thres. (**c**-**d**) The spatial activation of IC1 (**c**) and IC2 (**d**) estimated by FastICA. (**e**) The spatial activation of IC estimated by TCICA. (**f**) The activation detected by GLM

Figure 5 shows the group spatial activation of GLM and the group activation of the task-related components that were extracted by TCICA-Thres, FastICA and TCICA. Both TCICA-Thres and FastICA successfully extracted the two task-related components. The activation maps of the two task-related components largely overlapped with the two predefined ROIs (see Fig. 1B) for TCICA-Thres and FastICA (see Fig. 5A-D). However, TCICA only detected one task-related component, which mainly overlapped with the predefined ROI1 (see Fig. 5E). GLM detected the activated regions in both ROI1 and ROI2 (see Fig. 5F).

### The real fMRI experiment

**Fig. 6:**
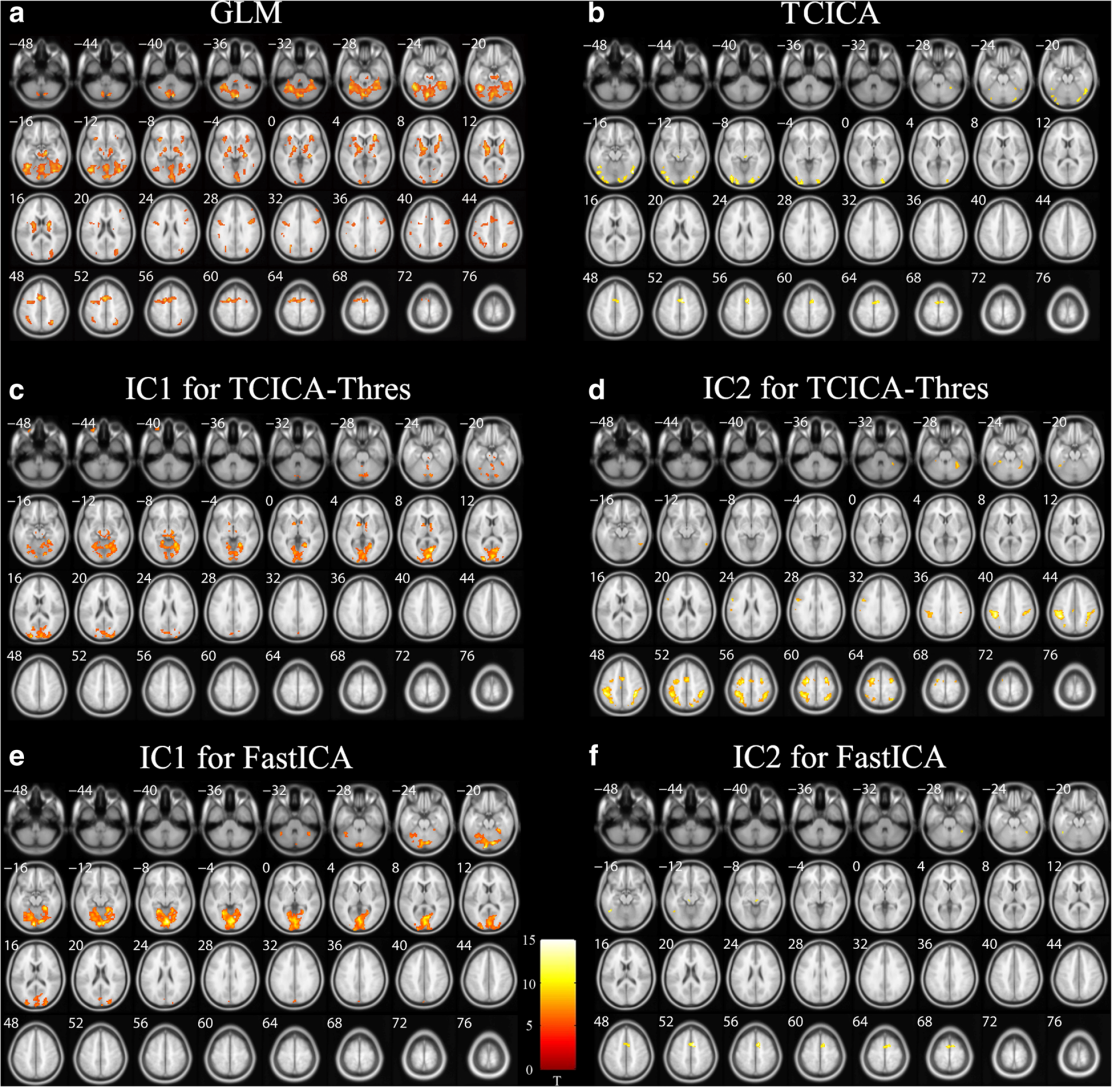
Group spatial activation of the fMRI data. (**a**) The activated regions detected by GLM. (**b**) The spatial activation of IC extracted by TCICA. (**c**-**d**) The spatial activation of IC1 (**c**) and IC2 (**d**) extracted by TCICA-Thres. (**e**-**f**) The spatial activation of IC1 (**e**) and IC2 (**f**) extracted by FastICA

Figure 6 shows the real fMRI data. The activated brain regions detected by GLM were mainly located in the parts of the visual cortex that were engaged in object perception and the parts of the motor cortex that were responsible for the motor output of judgment (see Fig. 6A). The activated visual cortex mainly included the middle occipital gyrus, the lingual gyrus and the fusiform gyrus. The activated motor cortex mainly contained the primary motor cortex, the premotor cortex, the supplementary motor cortex and the cerebellum. Two task-related components were extracted by TCICA-Thres and FastICA automatically. For TCICA-Thres and FastICA, the activation map of IC1 consisted of the visual cortex, and the activation map of IC2 consisted of the motor cortex (see Fig. 6C-F). For IC2, TCICA-Thres detected activation in the primary motor cortex, the premotor cortex, the supplementary motor cortex while FastICA only detected activation in the supplementary motor cortex. It can be inferred that IC1 was engaged in the visual processing and perception of objects and IC2 participated in the decision of finger tapping. Thus, the activated brain regions that were detected by GLM were distributed into the two task-related components that were extracted by TCICA-Thres and FastICA. It should be noted that the activated regions of IC2 for FastICA were much smaller than those for TCICA-Thres. For IC1, TCICA detected a smaller activated region in the visual cortex than FastICA. Moreover, TCICA only successfully separated one task-related component (see Fig. 6B). The activation map of the task-related component contained some visual cortex and a small supplementary motor cortex.

**Fig. 7:**
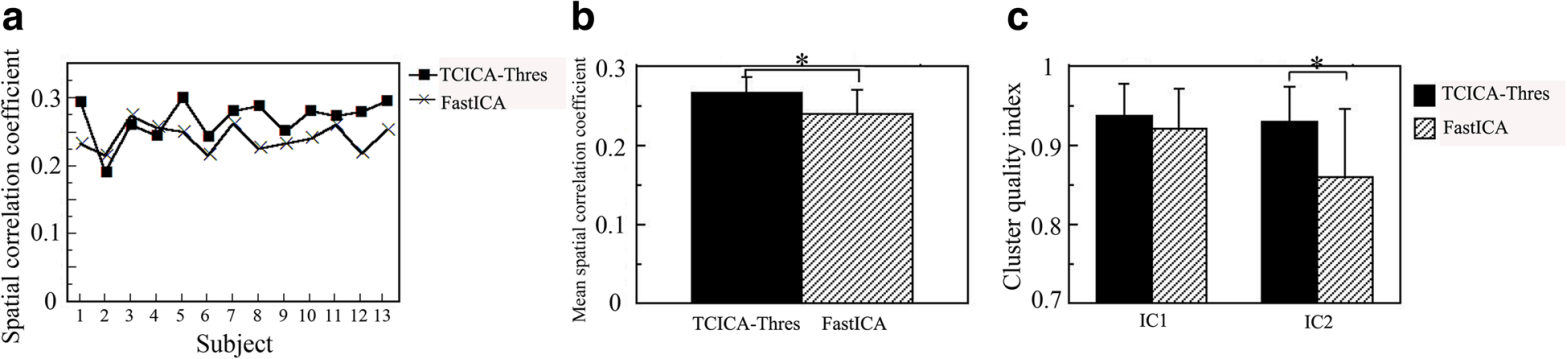
A comparison of TCICA-Thres and FastICA for fMRI data. (**a**) The spatial correlation coefficient of TCICA-Thres and FastICA for individual subjects. (**b**) The mean spatial correlation coefficients of TCICA-Thres and FastICA. (**c**) The mean cluster quality index of TCICA-Thres and FastICA. Note: asterisk represents *P* < 0.01

To compare the results that were estimated by GLM, TCICA-Thres and FastICA, we generated activation masks of the three methods. For GLM, the activation mask was created by setting the activated voxels to 1 and the non-activated voxels to 0. For TCICA-Thres and FastICA, the activation mask of IC1 was added to that of IC2 to generate one activation mask. The spatial correlation coefficients of the activation mask between GLM and TCICA-Thres/FastICA for all the subjects are shown in Fig. 7A. It can be seen that the TCICA-Thres result showed higher correlation with the GLM result than the FastICA result for all subjects except for subjects 2, 3 and 4. The means and standard deviations of the spatial correlation coefficients that were obtained by TCICA-Thres and FastICA are displayed in Fig. 7B. To further examine the differences in the spatial correlation coefficients between TCICA-Thres and FastICA, the nonparametric Wilcoxon test for paired samples was used to examine the difference between the two methods. The nonparametric Wilcoxon test indicated that TCICA-Thres results had significantly higher correlation with GLM results than with FastICA results (*p* < 0.01).

Figure 7C shows the mean of the cluster quality indices across all subjects of the two ICs extracted by Thres-ICA and FastICA. To examine the difference in the stability of the target IC estimation between the two methods, the nonparametric Wilcoxon test for paired samples was performed. The results showed that the stabilities of TCICA-Thres were significantly higher than FastICA for IC2 (*p* < 0.01).

## Discussion

In this study, we proposed the TCICA-Thres method that combined the TCICA method and the FastICA method through a threshold to automatically extract all the components related to the same task. The robustness and feasibility of the method under conditions of different noise levels and different temporal references were demonstrated, and the validity of the group TCICA-Thres method was confirmed. The results from both simulated and fMRI data suggest that TCICA-Thres was able to successfully extract all the task-related components and outperformed FastICA in spatial detection power and computation time.

In spite of the wide application of ICA to fMRI data, ICA needs to estimate all of the independent components from a dataset, which results in high computational-time costs and the requirement to select the desired components [17]. When TCICA is applied to fMRI data to extract the spatially independent components by adding the temporal constraint on mixing matrix, TCICA cannot successfully extract all the independent components that are related to the same task. For TCICA, the optimal surface of the cost function tends to merge all the extreme points that are close to the temporal reference into one extreme point due to the impact of the temporal constraint. As a result, the desired component that is extracted by TCICA method generally is the mixing of multiple task-related components. To separate all the task-related components automatically, TCICA-Thres combines the advantages of both TCICA and FastICA. TCICA-Thres sets a threshold to judge if the estimated parameter is close to the temporal reference. When the iteration is near the temporal reference, TCICA-Thres replaces the cost function of TCICA with the cost function of FastICA to keep all the extreme points close to the temporal reference. Otherwise, TCICA-Thres uses the cost function of TCICA to remove the extreme points that are far from the temporal reference. Therefore, TCICA-Thres can automatically extract all the components related to the same task without estimating all the components. Moreover, it should be noted TCICA-Thres is different from TSCICA, which was proposed in our previous study [10]. TCICA-Thres only uses the temporal constraint, while TSCICA uses both the temporal and spatial constraints.

The simulated data indicate that TCICA-Thres estimated all the independent components related to the task at all the noise levels. Compared to FastICA, TCICA-Thres showed higher spatial detection power at all noise levels for IC1 and at the middle noise levels for IC2 (see Fig. 2A and B). Moreover, TCICA-Thres took much less time to extract the desired components than FastICA (see Fig. 2C). These results suggest that TCICA-Thres had better robustness to noise and better computation efficiency than FastICA. Furthermore, TCICA-Thres kept a high performance and showed slight variations when CC between the temporal reference and the time course underlying the component was higher than 0.55 (see Fig. 3B). Moreover, the mean ROC area of TCICA-Thres dropped below 0.6 for CC less than 0.35. These results indicate that the TCICA-Thres method has a good robustness to the temporal reference. Because the temporal reference only helps TCICA-Thres remove the irrelevant extreme points, the performance of TCICA-Thres does not largely depend on the temporal reference. As long as the temporal reference shows a correlation with the task, TCICA-Thres can easily get rid of most extreme points that are unrelated to the task. When the iteration is close to the temporal reference, FastICA will help TCICA-Thres extract all the final task-related components. For the fMRI data, the temporal reference is usually derived from the convolution of the task paradigm with the ideal HRF. Although it is impossible to know the true HRF that underlies the fMRI data, the inaccuracy of temporal reference will not have much impact on the performance of TCICA-Thres in the fMRI data analysis. In this study, correlation that depends on delays may not be ideal to assess temporal match. The same oscillating frequency (perfectly related with task) with a little shift could give a lower correlation coefficient. In this study, TR (=2 s) was not adequate to sample task-related delays. The observed time course will be different from the true time course that drives each IC. Therefore, correlation is not ideal to measure the temporal match between the reference function and the time course underlying each IC.

The advantage of ICA over GLM is that ICA is powerful for identifying spatially distributed brain networks without any prior hypothesis regarding the data [2]. However, the TCICA-Thres and TCICA methods, which introduce temporal prior information into the ICA algorithm, are not purely exploratory anymore. Our simulated data indicate that the TCICA-Thres and FastICA method can successfully extracted all the target brain networks participating in the task from fMRI data, although the task activated two networks (see Fig. 4A-B). In contrast to FastICA, TCICA-Thres can automatically separate the task-related ICs without estimating all the components. Moreover, the brain network that was estimated by TCICA included the activated regions of both IC1 and IC2, which suggests that TCICA failed to efficiently dissociate the two task-related components. GLM identified all regions that were activated by the task, even when the estimated regions responded to two different time courses that were correlated to the same task. In contrast to GLM and TCICA, TCICA-Thres and FastICA detected much fewer falsely activated regions. Therefore, the simulated data demonstrated that TCICA-Thres had the strength of automatically separating all the brain networks that were engaged in the same task. Although GLM also detected the activated brain regions that overlapped with both ROI1 and ROI2, the activated regions that were detected by GLM were larger than those by TCICA. If there is one task-related component, it is easy for TCICA to find the extreme point that is close to the temporal reference. However, when fMRI data contain more than one components that are related to the same task, temporal reference may constrain TCICA in finding the extreme point that is close to the temporal reference. Because the task-related component that was extracted by TCICA was not the true task-related sources in fMRI data, it showed smaller activation than TCICA-Thres, FastICA and GLM.

Both simulated and real fMRI data demonstrated the feasibility of the group TCICA-Thres by combining TCICA-Thres with the temporal concatenation methods. For the simulated data, TCICA-Thres and FastICA successfully extracted two task-related ICs (see Fig. 5). The activated regions of IC1 and IC2 largely overlapped with the predefined ROI1 and ROI2 (see Fig. 1B). In contrast, TCICA only extracted one task-related IC, whose activated regions overlapped with ROI2 (see Fig. 5E). Moreover, GLM detected the activated regions in both ROI1 and ROI2 (see Fig. 5F). For the fMRI data, TCICA-Thres automatically estimated one task-related component that was engaged in visual processing and the other task-related component that was engaged in motor output. The two task-related components were also be identified by FastICA. Because the activated regions of IC1 and IC2 for TCICA-Thres and FastICA covered almost all the regions that were engaged in the task, the two methods successfully extracted all the ICs that were related to the task. In contrast, TCICA could only extract one task-related component, which consisted of most activated regions of IC1 and a few activated regions of IC2. Although the activated regions that were detected by TCICA-Thres and FastICA largely overlapped with the regions that were estimated by GLM, the activated motor cortex of IC2 that was estimated by TCICA-Thres was much larger than that by FastICA. As a result, the activation pattern of TCICA-Thres showed a higher correlation with that of GLM than it did with FastICA. In addition, TCICA-Thres showed better stability than FastICA, especially for IC2. The results from the real fMRI experiment further verify that TCICA-Thres outperformed TCICA and FastICA in spatial detection power.

To avoid an endless loop, the learning rate of optimization algorithms is usually set to decrease as the iterative step increases. Because TCICA-Thres needs to switch the cost function between TCICA and FastICA during the iteration, the learning rate cannot be of exponential form, which would decrease too rapidly with the iterative step. In this study, the learning rate in TCICA-Thres was set as 10^4^ × (0.5 × cos(pi×(k-1)/99) + 0.5)^n^. The learning rate used in this study decreased slowly at the beginning of iteration, rapidly in the middle of iteration and slowly again at the end of iteration. The parameter *n*, which controlled the decreasing speed of the learning rate, was determined by the simulated data. In this study, we chose *n* = 2 as the optimal value according to the ROC results from the simulation. Moreover, the mean ROC area varied slightly when *n* varied from 1 to 4 (see Fig. 2D). Thus, the results would be stable for *n* ranging from 1 to 4, although 2 was selected as the optimal value of *n* in the study. The results from both simulated and fMRI data confirm that this rule worked well.

TCICA-Thres showed good stability for all the simulated and real datasets, except for the fMRI data of one subject. When applying TCICA-Thres to this subject, the algorithm went back and forth rapidly between constrained TCICA and FastICA in many iterative steps. Although the algorithm showed frequent rapid switches between TCICA and FastICA, the iteration finally stabilized in the FastICA stage. Because the learning rates became very slow after many switches, the TCICA-Thres algorithm could not converge within the maximum 100 iteration steps. To avoid the slow learning rate and solve the nonconvergence issue, we reset the iteration step to 1 and reset the unmixing matrix W to the value of the iteration that switched from TCICA to FastICA the last time after the TCICA-Thres algorithm stabilized in the FastICA stage. The criterion that was used to judge if the iteration of TCICA-Thres stabilized in FastICA was if the iteration time of FastICA was larger than 5. After such processing, the TCICA-Thres algorithm estimated the stable task-related components from the fMRI data of the subject.

The essence of TCICA-Thres is to introduce the temporal constraint when the correlation coefficient *ρ* between the unmixing vector *w* and ${r}_t^{\hbox{'}}$ , where ${r}_t^{\hbox{'}}$ is the transformation of the temporal reference *r*_t_ into the unmixing space, is smaller than the *threshold*. For the TCICA-Thres method, the *threshold* of *ρ* is an important parameter that is similar to the weight between the TCICA part and the FastICA part. A bigger *ρ* indicates a higher weight of TCICA and a smaller weight of FastICA. However, it is impossible in most applications to obtain accurate prior temporal information in fMRI data before ICA processing. Therefore, a bigger *threshold* does not mean better results. On the other hand, if the *threshold* is too small, more irrelevant components may be included during FastICA iteration for TCICA-Thres. Taking these aspects into consideration, the *threshold* of ρ was set to an empirical value of 0.5, which meant that TCICA and FastICA had the same weight in the TCICA-Thres algorithm. Moreover, because the number of components that are related to a task in fMRI data is unknown, the *threshold* is also used to determine whether the extracted IC is related to the task. The estimated component was considered a task-related component when the correlation coefficient between the temporal reference and the time course of IC was greater than 0.5. Otherwise, the TCICA-Thres algorithm will terminate. The TCICA algorithm is terminated when the correlation between the time course of IC and the temporal reference is lower than 0.5. The results from both simulated and real fMRI experiments demonstrate that the value of *threshold* works well.

It should be noted that there are some limitations to the current study. First, the *threshold* of the correlation coefficient ρ was set to an empirical value of 0.5. It is worthwhile to investigate an optimal ρ in the future. Second, the proposed TCICA-Thres method cannot be used in the resting fMRI data because the resting data do not have temporal prior information.

## Conclusions

We demonstrated the feasibility and robustness of the TCICA-Thres method, which incorporated both TCICA and FastICA methods by using a threshold. The performances of the proposed method and FastICA were compared using both simulated and fMRI data. The results indicate that TCICA-Thres is capable of automatically extracting all the task-related components and has better spatial detection power, computation efficiency and robustness to noise than FastICA. Moreover, TCICA-Thres displays good robustness to temporal prior information.

## Additional file

Additional file 1: **Appendix.** Details of the TCICA-Thres algorithm. (DOCX 22 kb)

## Abbreviations

CC: correlation coefficient
CICA: constrained ICA
CNR: contrast-to-noise ratios
EPI: echo planar imaging
fMRI: functional magnetic resonance imaging
FOV: field of view
GLM: general linear model
HRF: hemodynamic response
ICA: independent component analysis
ICs: independent components
MDL: minimum description length
PCA: Principle component analysis
ROC: receiver operation characteristic
ROI: Region of Interest
TCICA: temporally constrained independent component analysis
TCICA-Thres: TCICA with threshold
TE: echo time
TR: repeated time
TSCICA: temporally and spatially constrained ICA

## References

[1] Hyvarinen A, Oja E (2000). Independent component analysis: algorithms and applications. Neural Netw.

[2] Mckeown MJ, Makeig S, Brown GG, Jung TP, Kindermann RS, Bell AJ, Sejnowski TJ (1998). Analysis of fMRI data by blind separation into independent spatial components. Hum Brain Mapp.

[3] Ma X, Zhang H, Zhao X, Yao L, Long Z (2013). Semi-blind independent component analysis of fMRI based on real-time fMRI system. IEEE Transactions on Neural Systems and Rehabilitation Engineering.

[4] Calhoun V, Adali T, Stevens M, Kiehl K, Pekar J (2005). Semi-blind ICA of fMRI: a method for utilizing hypothesis-derived time courses in a spatial ICA analysis. Neuroimage.

[5] Lu W, Rajapakse JC (2005). Approach and applications of constrained ICA. IEEE Trans Neural Netw.

[6] Lu W, analysis RJCC i c. In in advances in neural information processing systems 13 (NIPS2000). Citeseer. 2000:570–6.

[7] Sun ZL, Shang L (2010). An improved constrained ICA with reference based unmixing matrix initialization. Neurocomputing.

[8] Lin QH, Liu J, Zheng YR, Liang H, Calhoun VD (2010). Semiblind spatial ICA of fMRI using spatial constraints. Hum Brain Mapp.

[9] Wang Z (2011). Fixed-point algorithms for constrained ICA and their applications in fMRI data analysis. Magn Reson Imaging.

[10] Wang Z, Xia M, Jin Z, Yao L, Long Z (2014). Temporally and spatially constrained ICA of fMRI data analysis. PLoS One.

[11] Rodriguez PA, Anderson M, Calhoun VD, Adali T (2015). General nonunitary constrained ICA and its application to complex-valued fMRI data. IEEE Trans Biomed Eng.

[12] Hyvarinen A (1999). Fast and robust fixed-point algorithms for independent component analysis. IEEE Trans Neural Netw.

[13] Independent Component Analysis (ICA) and Blind Source Separation (BSS). http://www.cis.hut.fi/projects/ica/fastica/. Accessed 5 March 2013.

[14] Allen AE, Erhardt EB, Wei Y, Eichele T, Calhoun VD. A simulation toolbox for fMRI data: SimTB. http://mialab.mrn.org/software/simtb/index.html. Accesed 24 June 2011. 10.1016/j.neuroimage.2011.11.088 PMC3690331 22178299

[15] Statistical Parametric Mapping software. http://www.fil.ion.ucl.ac.uk/spm/software/. Accesed April 2009.

[16] Oldfield RC (1971). The assessment and analysis of handedness: the Edinburgh inventory. Neuropsychologia.

[17] Luo J, Hu B, Ling XT, Liu RW (1999). Principal independent component analysis. Neural Networks IEEE Transactions on.

