# Supplementary material for: Temporally constrained ICA with threshold and its application to fMRI data
Source: BMC Med Imaging. 2019 Jan 17;19:6. doi: 10.1186/s12880-018-0300-6 (PMC6337805; doi:10.1186/s12880-018-0300-6)
Supplement: Supplementary file 1 — Appendix. Details of the TCICA-Thres algorithm. (DOCX 22 kb) [file 12880_2018_300_MOESM1_ESM.docx]

Appendix

A gradient descent learning algorithm can be used to solve the optimization problem in Eq. (6).

$w_{k+1}=w_{k}-\eta\nabla_{w}L$ (A1)

$\nabla_{w}L=\left\{ \begin{aligned} E\left\{ \hat{\mathbf{X}}f^{'}\left( w_{k}^{T}\hat{\mathbf{X}} \right) \right\}-s_{c}\mu g^{'}\left( w_{k}, r_{t}^{'} \right)-\lambda w_{k}, \rho\left( w_{k}, r_{t}^{'} \right)\leq threshold \\ E\left\{ \hat{\mathbf{X}}f^{'}\left( w_{k}^{T}\hat{\mathbf{X}} \right) \right\}-\lambda w_{k}, \rho\left( w_{k}, r_{t}^{'} \right)>threshold \end{aligned} \right.$ (A2)

The optimum multipliers *μ* and λ are iteratively updated based on following equation:

$\mu_{k+1}=\max(0,\mu_{k}+\gamma g(w_{k},r_{t}^{'}))$ (A3)

$\lambda_{k+1}=E\{w_{k}^{T}\hat{\mathbf{X}}f'(w_{k}^{T}\hat{\mathbf{X}}$ (A4)

The learning rate η was set to 10^-4^×(0.5×cos(π×(k-1)/99)+0.5)^n^ that decreased with the iterative step k.

If there are several task-related components in the data, the TCICA-Thres method can only estimate one of the task-related components in one TCICA-Thres separation. To estimate all the task-related components and prevent different vectors from converging to the same maxima, we must decorrelate the outputs after every iteration. When we have extracted *p* (*p*≥1) task-related components, or *p* vectors *w*_1_,… ,*w_p_*, we run TCICA-Thres method for *w_p+1_*. After every iteration step, *w_p+_*_1_ is decorrelated and normalized as follows:

$w_{p+1}=w_{p+1}-\sum_{j=1}^{p} w_{p+1}^{T}w_{j}w_{j}$ (A5)

$w_{p+1}^{*}=w_{p+1}/||w_{p+1}||$ (A6)

The termination criterion of the TCICA-Thres is set to be ||Δ*w*||<10^-4^ in each component’s separation. After separating one component, the correlation coefficient (CC) between the temporal reference and the time course of the estimated component is calculated. If CC is larger than *threshold,* the output is considered a desired task-related component, and the next component is extracted sequentially. Otherwise, the output is discarded, and the TCICA-Thres algorithm is terminated immediately.

The procedure of TCICA-Thres is listed as follows:

Step 1: Center and whiten the observed signal **X.**

Step 2: Initialize *γ*, *μ, ξ* and *λ***;** set *threshold=*0.5.

Step 3: Initialize *w_i_* as a random vector. Center and normalize *w_i_***.**

Step 4: For the *k*th iterative step.

Step 5: Update *w_i_* using Eq. (A1) and (A2).

Step 6: Update *ξ* and *λ* using Eq. (A3) and (A4).

Step 7: Decorrelate *w_i_* using Eq. (A5) and normalize *w_i_* using Eq. (A6).

Step 8: Jump to step 4 and repeat the step 5 to 7 until ||Δ*w_i_*||<10^-4^ or k>100.

Step 9: When the iteration stops, the independent component can be obtained according to *y=w*^T^**X**.

Step 10: Calculate the value of the CC between the temporal reference and the time course of the extracted component.

If the CC is larger than *threshold*, the output is considered a task-related component, and the algorithm jumps to step 2.

Otherwise, the algorithm is terminated immediately.
